# Simulation study on LDL cholesterol target attainment, treatment costs, and ASCVD events with bempedoic acid in patients at high and very-high cardiovascular risk

**DOI:** 10.1371/journal.pone.0276898

**Published:** 2022-10-27

**Authors:** Julius L. Katzmann, Christian Becker, Aikaterini Bilitou, Ulrich Laufs

**Affiliations:** 1 Klinik und Poliklinik für Kardiologie, Universitätsklinikum Leipzig, Leipzig, Germany; 2 Daiichi Sankyo Deutschland GmbH, München, Germany; 3 Daiichi Sankyo Europe GmbH, München, Germany; Medizinische Hochschule Hannover, GERMANY

## Abstract

**Background and aims:**

The LDL cholesterol (LDL-C) treatment goals recommended by the 2019 ESC/EAS guidelines are only achieved in a minority of patients. The study objective was to estimate the impact of bempedoic acid treatment on LDL-C target attainment, drug costs, and atherosclerotic cardiovascular disease (ASCVD) events.

The simulation used a Monte Carlo approach in a representative cohort of German outpatients at high or very-high cardiovascular risk. Additionally to statins, consecutive treatment with ezetimibe, bempedoic acid, and a PCSK9 inhibitor was simulated in patients not achieving their LDL-C goal. Considered were scenarios without and with bempedoic acid (where bempedoic acid was replaced by a PCSK9 inhibitor when LDL-C was not controlled).

**Results:**

The simulation cohort consisted of 105,577 patients, of whom 76,900 had very-high and 28,677 high cardiovascular risk. At baseline, 11.2% of patients achieved their risk-based LDL-C target. Sequential addition of ezetimibe and bempedoic acid resulted in target LDL-C in 33.1% and 61.9%, respectively. Treatment with bempedoic acid reduced the need for a PCSK9 inhibitor from 66.6% to 37.8% and reduced drug costs by 35.9% per year on stable lipid-lowering medication. Compared to using only statins and ezetimibe, this approach is projected to prevent additional 6,148 ASCVD events annually per 1 million patients, whereas PCSK9 inhibition alone would prevent 7,939 additional ASCVD events annually.

**Conclusions:**

A considerably larger proportion of cardiovascular high- and very-high-risk patients can achieve guideline-recommended LDL-C goals with escalated lipid-lowering medication. Bempedoic acid is projected to substantially decrease the need for PCSK9 inhibitor treatment to achieve LDL-C targets, associated with reduced drug costs albeit with fewer prevented events.

## 1 Introduction

The treatment goals for low-density lipoprotein cholesterol (LDL-C) are only achieved in a minority of patients at high and very-high cardiovascular risk [1,2]. Based on healthy lifestyle, the current recommendations of the European Society of Cardiology and the European Atherosclerosis society (ESC/EAS) include a stepwise approach of treatment with statins, ezetimibe, and monoclonal antibodies against PCSK9 to attain LDL-C targets [3]. PCSK9 inhibitors (PCSK9i) are highly effective in reducing LDL-C and have a favourable side effect profile. However, partly due to their high cost, their use is limited and subject of reimbursement restrictions [4].

Recent clinical recommendations stress the need for early oral combination lipid-lowering therapy (LLT) [5]. The orally administered ATP citrate lyase inhibitor bempedoic acid reduces LDL-C in patients on statin by ~18%–25% as single agent on top of background LLT [6] and by ~38% as a combination with ezetimibe [7]. Bempedoic acid has the potential to reduce the need of PCSK9i treatment in patients not attaining the LDL-C target while on a statin and/or ezetimibe.

The cardiovascular outcome trial with bempedoic acid, CLEAR Outcomes [8], is ongoing. With definitive results pending, simulation studies may provide guidance on the effectiveness of bempedoic acid in reducing LDL-C, treatment costs, and cardiovascular outcomes. A recent, single-centre simulation study in patients with coronary artery disease found that implementing bempedoic acid as adjunct LLT in the treatment algorithm would reduce the need for PCSK9i treatment and consequently drug costs to attain LDL-C targets [9]. However, current representative data of nationwide high- and very-high-risk patients are lacking.

Using anonymized, real-world data from the IQVIA^TM^ Disease Analyzer [10], we investigated to which extent bempedoic acid treatment may reduce the need of PCSK9i treatment, drug costs, and atherosclerotic cardiovascular disease (ASCVD) events in Germany. We applied a Monte Carlo approach simulating scenarios with and without bempedoic acid in a representative cohort of high- and very-high-risk patients.

## 2 Patients and methods

This cohort study was conducted using data from the IQVIA^TM^ Disease Analyzer that is representative for the German population with respect to age, gender, prescription patterns, and chronic diseases such as cancer, dementia, and diabetes [10,11]. The database contains anonymized data from statutory and privately insured patients from a panel of more than 3,300 ambulatory general practitioners (GPs) and specialists in Germany and allows for longitudinal analyses of real-world diagnostic and therapeutic behaviour. This study did not require ethical approval, as only anonymized data were obtained and analysed.

### 2.1 Study period and study population

**Fig 1:**
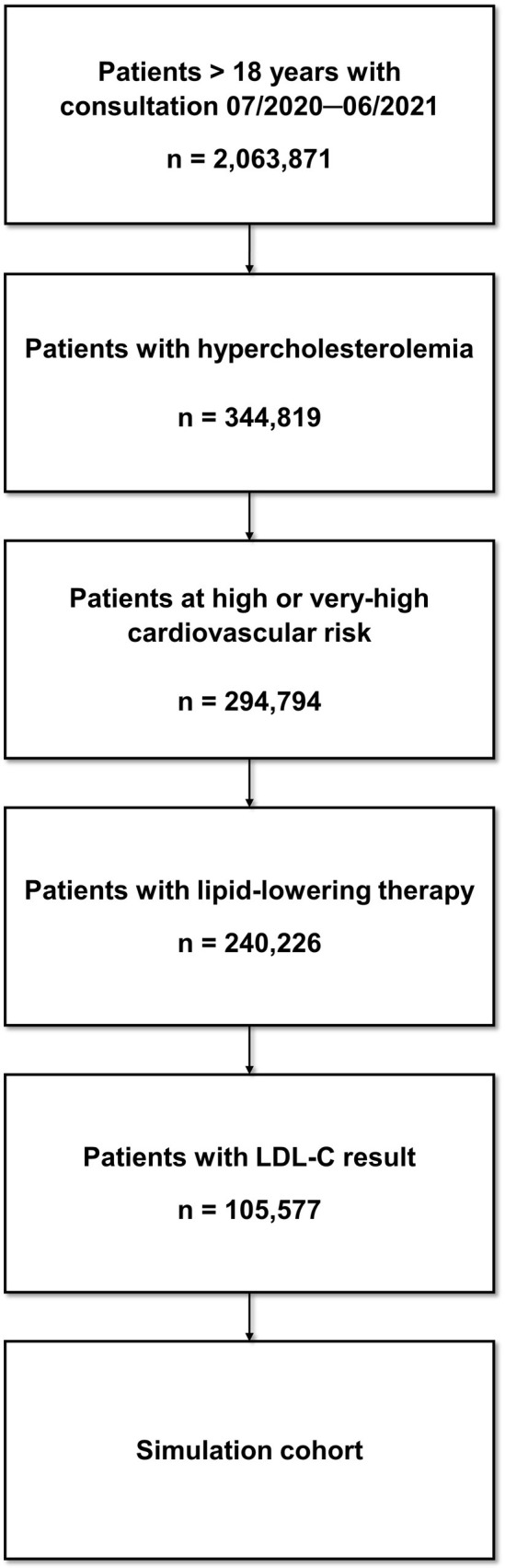
Flow chart of patient selection.

The study period was defined as July 2020 to June 2021. Patients were included if they met the following criteria (**Fig 1**).

- consultation within the study period
- age of at least 18 years at index date
- hypercholesterolemia (based on diagnosis or LLT prescription)
- high or very-high cardiovascular risk according to the 2019 ESC/EAS guidelines [3]
- prescription of LLT
- available LDL-C result

The index date was defined by the latest date of an LDL-C result within the study period. Statin intensity was defined as previously described (**S1 Table**) [12]. Baseline characteristics, including laboratory parameters and components of the SCORE (Systematic Coronary Risk Estimation [3]) were assessed at the index date based on a look-back period of 12 months. ASCVD and diabetes mellitus were assumed to be present if they had been diagnosed at any time in the past. Chronic kidney disease was defined by glomerular filtration rate or respective ICD 10 codes within 60 months prior to index date. The ICD 10 codes underlying the diagnoses are detailed in **S2 Table**.

LLT was defined as statins, ezetimibe, bempedoic acid, PCSK9i including inclisiran, and fixed-dose combinations of these drugs. LDL-C results were used if LLT was prescribed at least 4 weeks prior. Outlier laboratory results (~0.1% of the lower and upper values) were excluded. The LDL-C treatment targets were defined in accordance with the current ESC/EAS guidelines as < 55 mg/dL for very-high and < 70 mg/dL for high-risk patients [3].

### 2.2 Simulation of LLT

**Fig 2:**
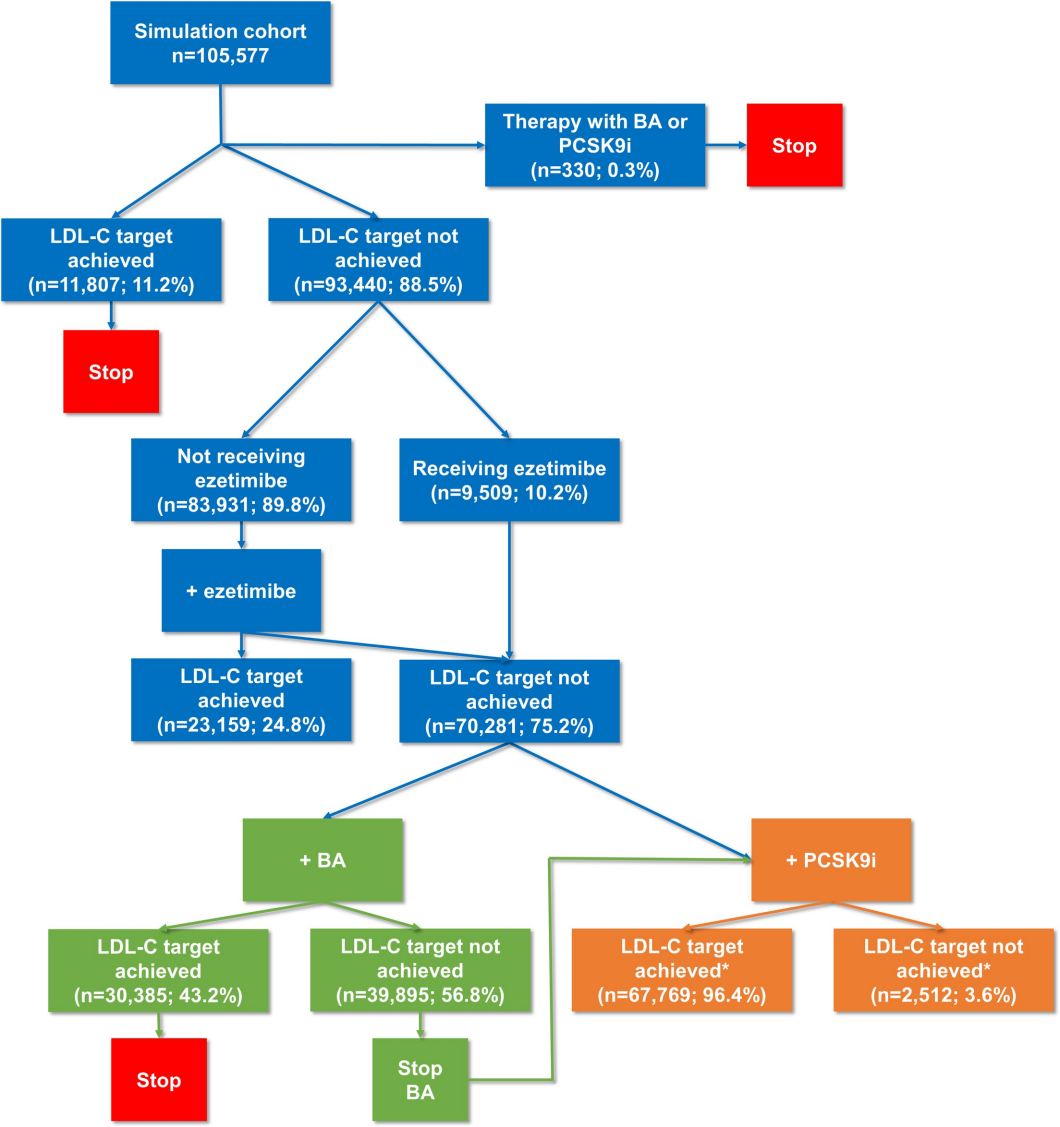
Treatment algorithm applied in the simulation study. Scenario (1) is shown in green, scenario (2) in orange. Percentages refer to the prior step of the algorithm. Slightly different patient numbers between consecutive steps of the simulation algorithm are the result of summarizing the counts of respective patients across the 10,000 simulations by the median. Notes: * Numbers of patients achieving the LDL-C goal are provided for scenario (2). The numbers slightly differed for scenario (1) with n = 67,853 (96.5%) of patients achieving the LDL-C target and n = 2,428 (3.5%) not. BA: Bempedoic acid, PCSK9i: PCSK9 inhibitor, LDL-C: Low-density lipoprotein cholesterol.

The simulations were performed using a Monte Carlo approach with probabilistic simulation of treatments effects as in previous analyses [9,13]. The treatment algorithm included sequential simulation steps of LLT add-on (ezetimibe or bempedoic acid) performed in patients not at their LDL-C goal with their existing treatments. No statin intensification was simulated; patients were assumed to be on their maximum tolerated statin at index. Patients with PCSK9i or bempedoic acid treatment at index date did not enter the simulation model and kept their LDL-C results. The addition of ezetimibe was simulated in patients not on ezetimibe and not at the LDL-C goal at baseline, irrespective of statin treatment and intensity. Secondly, two different scenarios were considered in patients not at goal after ezetimibe treatment. In scenario (1), the addition of bempedoic acid was simulated. If the LDL-C target was not achieved with bempedoic acid, bempedoic acid was stopped and PCSK9i treatment effect was added. In scenario (2), patients not at target after the addition of ezetimibe received PCSK9i without simulating bempedoic acid treatment (constituting the scenario without bempedoic acid; **Fig 2**).

The effect of ezetimibe on LDL-C was simulated probabilistically sampled from a beta distribution as in a previous simulation study [13] and based on published data [14,15] (mean decrease 22.9%, standard deviation [SD] 14.8%). For the simulation of the effect of bempedoic acid on LDL-C, patient-level data of the CLEAR phase 3 studies were used (observed values of 12 week LDL-C reduction from baseline), stratified by statin intensity [16–19] (moderate- or high-dose statins: mean [SD] 16.7% [20.9%], low-dose or no statin: 24.1% [22.3%]). The LDL-C reduction by PCSK9i treatment was assumed as 59% [9,20].

Probabilistic sampling was run 10,000 times on the complete set of patients. At each of the 10,000 runs, the mean LDL-C value of the cohort was calculated and of these 10,000 mean values, the median was derived.

### 2.3 Drug costs and effect on cardiovascular events

The annual drug costs were based on current pharmacy sales price including VAT in Germany for the drug packages associated with the lowest drug cost per day (retrieved from Lauer-Taxe® January 1^st^, 2022) as follows: Bempedoic acid/ezetimibe fixed-dose combination 968.70 Euro, and evolocumab 5,735.48 Euro. Drug costs were calculated per 1 million patients.

The primary endpoint of major adverse cardiac events (4P-MACE) was defined in accordance with the ongoing CLEAR Outcomes trial [8] as composite of cardiovascular death, non-fatal myocardial infarction, non-fatal stroke, or coronary revascularization (**S3 Table**).

The baseline 4P-MACE rate was defined as the first occurrence of any of the 4P-MACE components within 12 months of the index date. To allow for 12 full months after index, for the purpose of this calculation, patients were selected in a study period between July 2019 and June 2020. The relative risk reductions were calculated for each LLT scenario based on the median LDL-C reduction of the whole simulation cohort. The relative risk reductions per 1 mmol/L (38.67 mg/dL) LDL-C reduction were based on the 2010 and 2015 CTTC meta-analyses of statin trials (e.g., for the composite endpoint, 21% relative risk reduction per mmol/L LDL-C reduction) [21,22]. Finally, the number of additional annually prevented events per 1 million patients compared to using only statins and ezetimibe and the drug costs per prevented event were calculated.

### 2.4 Statistical analyses

The statistical analyses were performed with SAS 9.4 (SAS Institute Inc., Cary, North Carolina, USA) and R version 4.1.0 with the packages haven (version 2.4.3) and MonteCarlo (version 1.0.6).

## 3 Results

### 3.1 Simulation cohort

Within the study period, 2,063,871 patients consulted a GP or cardiologist. Of these patients, 105,577 fulfilled all inclusion criteria and represented the simulation cohort. The flow chart of patient selection is depicted in **Fig 1**.

Of the total simulation cohort, 76,900 patients had very-high and 28,677 had high cardiovascular risk. The baseline characteristics for the total cohort and for the two cardiovascular risk strata are shown in **Table 1**. The mean age was 70.7 years, 42.9% were female. Coronary artery disease was present in 49.7%, cerebrovascular disease in 13.4%, and peripheral vascular disease in 24.3%. The most frequent cardiovascular risk factor was hypertension in 84.7% of patients. Mean LDL-C at baseline was 92.1 mg/dL, 11.2% of patients achieved their LDL-C target. The most frequent LLT was statin monotherapy (88.3%), with most patients receiving moderate intensity statin. Less than one in ten patients received a statin-ezetimibe combination (9.9%).

**Table 1:** Baseline characteristics.

	Total cohort	Very-high cardiovascular risk	High cardiovascular risk
**General**			
**N**	105,577	76,900	28,677
**Female (%)**	42.9	38.7	54.0
**Age (mean [SD] in years)**	70.7 (11.0)	71.8 (10.8)	67.8 (11.2)
**Body mass index (mean [SD] in kg/m**^**2**^)	29.2 (5.4)	29.1 (5.3)	29.6 (5.5)
**Atherosclerotic cardiovascular disease** ^**a**^			
**Coronary artery disease (%)**	49.7	68.3	0
**Cerebrovascular disease (%)**	13.4	18.4	0
**Peripheral artery disease (%)**	24.3	33.4	0
**Cardiovascular risk factors**			
**Hypertension (%)**	84.7	84.4	85.3
**Diabetes (%)**	54.0	57.1	45.7
**Current smoking (%)**	48.1	46.4	54.9
**Lipids**			
**LDL cholesterol (mean [SD] in mg/dL)**	92.1 (31.5)	88.3 (30.0)	102.3 (32.8)
**LDL cholesterol at target (%)**^**b**^	11.2	10.0	14.2
**Lipid-lowering medication**			
**Statin monotherapy (%)**	88.3	86.3	93.4
**Low intensity (%)**	4.2	3.8	5.4
**Moderate intensity (%)**	61.6	57.8	71.7
**High intensity (%)**	22.5	24.8	16.3
**Ezetimibe monotherapy (%)**	1.5	1.6	1.4
**Statin + ezetimibe (%)**	9.9	11.7	5.1
**Other lipid-lowering therapies (%)**	0.3	0.4	0.1

Notes: All percentages refer to non-missing values.

^a^ Definition of very-high cardiovascular risk includes history of atherosclerotic cardiovascular disease.

^b^ Including patients who received bempedoic acid or PCSK9 inhibitor at baseline.

SD: Standard deviation, LDL: Low-density lipoprotein.

Patients with high compared to patients with very-high cardiovascular risk were slightly younger, had a higher female proportion, and a higher LDL-C (mean [SD] 102.3 [32.8] mg/dL compared to 88.3 [30.0] mg/dL). A higher proportion of high-risk patients (14.2%) achieved their LDL-C goal of < 70 mg/dL at baseline, while only 10.0% of very-high-risk patients attained their 55 mg/dL goal. The majority of high-risk patients received statin monotherapy (93.4%) and less combination therapy compared to very-high-risk patients.

### 3.2 Simulation of bempedoic acid and PCSK9i treatment

Within the simulation cohort of n = 105,577 patients, 0.3% of patients received bempedoic acid or PCSK9i at baseline and did not enter the simulation algorithm. Of the remaining patients, 88.5% did not achieve their LDL-C target and entered the first simulation step of treatment with ezetimibe, if they were not already on ezetimibe treatment. After simulation, 24.8% of the patients achieved their LDL-C target (cumulative related to the whole simulation cohort: 33.1%).

**Fig 3:**
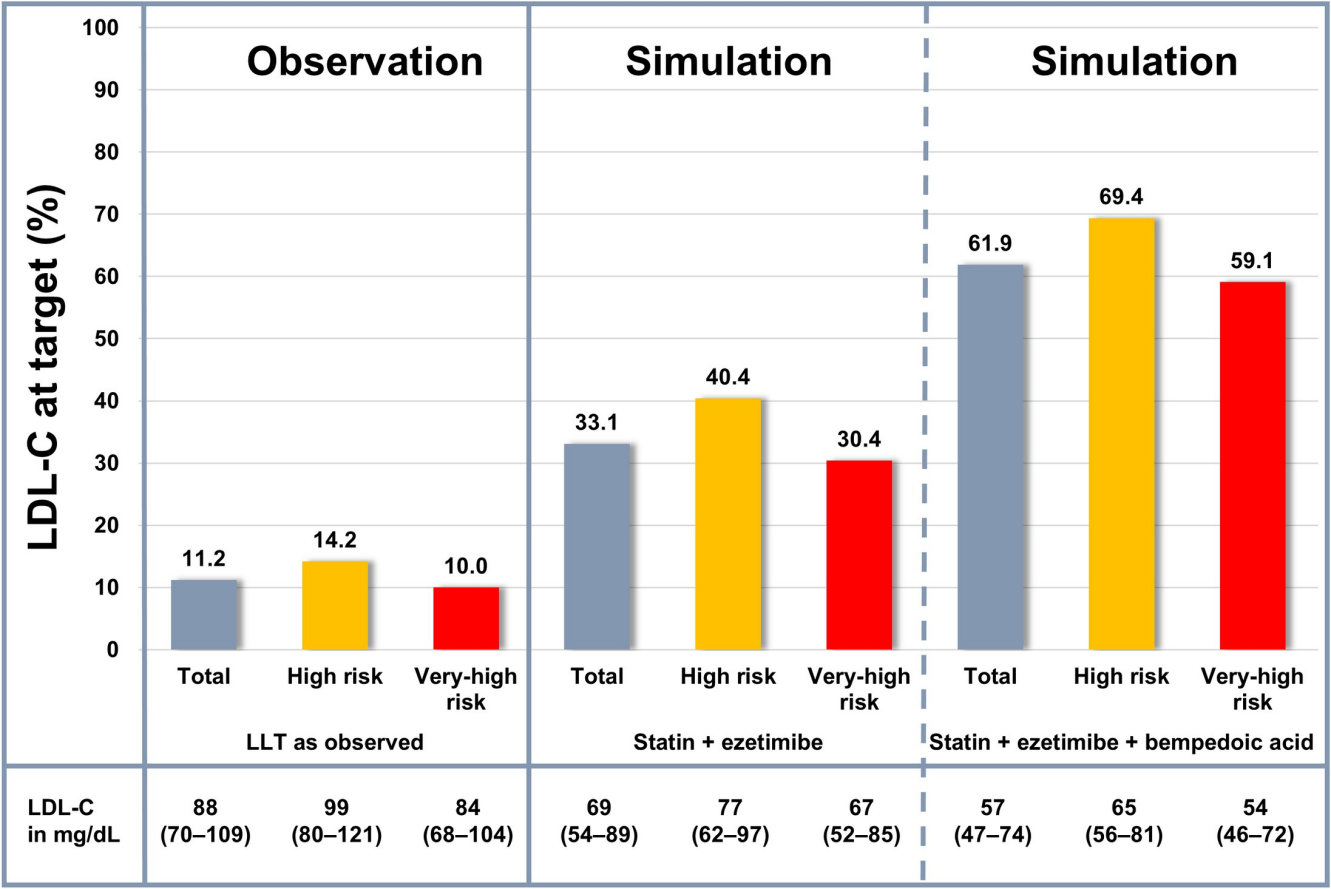
LDL-C target attainment in the total cohort and stratified by risk as observed and simulated. LDL-C concentrations are given as median (interquartile range). LDL-C: Low-density lipoprotein cholesterol. LLT: Lipid-lowering therapy.

Patients still not at LDL-C goal entered two possible scenarios:

In scenario (1), the addition of bempedoic acid was simulated. Out of patients with previously uncontrolled LDL-C on statins and ezetimibe, 43.2% reached their LDL-C goal upon bempedoic acid treatment (cumulative related to the whole simulation cohort: 61.9%). The proportion of patients at LDL-C goal after bempedoic acid was higher in high-risk compared to very-high-risk patients (69.5% vs. 59.1% of the total cohort; **Fig 3**). In patients who did not reach the goal after the simulation of bempedoic acid treatment, bempedoic acid was stopped and PCSK9i initiated as per clinical opinion. Afterwards, 96.5% of patients had controlled LDL-C (cumulative related to the whole simulation cohort: 97.7%).

In scenario (2), patients were simulated receiving a PCSK9i without prior bempedoic acid treatment. In these patients, the LDL-C target was achieved in 96.4% (cumulative related to the whole simulation cohort: 97.6%). The two scenarios and the proportion of patients at LDL-C goal at each step of the algorithm are depicted in **Fig 2**.

Treatment with bempedoic acid as simulated in scenario (1) reduced the need for treatment with a PCSK9i as in scenario (2) from 66.6% to 37.8% of the total cohort.

### 3.3 Treatment costs and cardiovascular events

Add-on treatment with bempedoic acid to statins and ezetimibe reduced the need of PCSK9i treatment in scenario (1) by 28.8% in the simulation cohort. This resulted in annual reductions of drug costs of 1.37 billion Euro per one million treated patients on stable LLT (35.9%; **Table 2**).

**Table 2:** Drug costs, prevented atherosclerotic cardiovascular disease events, and cost per prevented event.

Simulation add-on scenarios after statins and ezetimibe	BA/PCSK9i scenario (1)	PCSK9i scenario (2)	Difference scenarios (2)-(1)
**Proportion of patients achieving goal after BA treatment (%)**	28.8	–	–
**Proportion of patients requiring PCSK9i treatment to achieve goal (%)**	37.8	66.6	28.8
**Drug costs BA per 1 million patients/year (€)**	278,791,304	–	-278,791,304
**Drug costs PCSK9i per 1 million patients/year (€)**	2,167,299,455	3,818,021,632	1,650,722,177
**Drug cost combined (€)**	2,446,090,759	3,818,021,632	1,371,930,873 (35.9%)
**LDL-C reduction**^**a**^ (mg/dL)	23.7	31.3	7.6
**Achieved LDL-C (median [IQR])**	45.5	37.9	-7.6
**LDL-C at target (%)**	97.7	97.6	-0.1
**Relative risk reduction (%)**	13.5	17.4	3.9
**Prevented events annually per 1 million treated patients**			
**Total**	6,148	7,939	1,791 (22.6%)
**Cardiovascular death**	1	1	0
**Myocardial infarction**	4,593	5,896	1,303
**Stroke**	1,394	1,812	418
**Coronary revascularization**	1,224	1,575	351
**Drug cost per prevented event annually (€)**			
**Total**	397,838	480,900	83,062 (17.3%)
**Cardiovascular death**	3,094,982,802	3,703,402,897	608,420,095
**Myocardial infarction**	532,516	647,582	115,066
**Stroke**	1,754,681	2,106,543	351,862
**Coronary revascularization**	2,423,818	1,998,015	425,803

Notes: BA: Bempedoic acid, PCSK9i: PCSK9 inhibitor, LDL-C: Low-density lipoprotein cholesterol, IQR: Interquartile range.

Annual drug costs according to current pricing: Fixed-dose combination BA/ezetimibe: 968.70 Euro, PCSK9 inhibitor: 5,735.48 Euro.

^a^Compared to using only statins and ezetimibe.

The use of bempedoic acid in scenario (1) led to absolute LDL-C reductions of 23.7 mg/dL with an achieved median LDL-C of 45.5 mg/dL. The corresponding relative risk reduction for the 4P-MACE endpoint was 13.5%. The absolute LDL-C reductions were larger in scenario (2) with an absolute decrease of 31.3 mg/dL, a median achieved LDL-C of 37.9 mg/dL, and a corresponding relative risk reduction of 17.4%.

The baseline annual event rate estimated in the cohort was 4.6%. Per one million patients, in scenario (1), 6,148 first 4P-MACE were prevented compared to 7,939 prevented first events in scenario (2). The components of the primary endpoint are detailed in **Table 2**.

The annual incremental drug cost per prevented first MACE per one million patients was 397,838 Euro in scenario (1) compared to 480,900 Euro in scenario (2), corresponding to a relative difference of 17.3%. The differences in drug costs for the prevention of the endpoint components are detailed in **Table 2**.

## 4 Discussion

The present study has two main findings. First, the simulations show that a large proportion of cardiovascular high- and very-high-risk patients can achieve guideline-recommended LDL-C targets with oral LLT with a stepwise approach of statin, ezetimibe, and bempedoic acid. Secondly, the strategy of implementing bempedoic acid prior to PCSK9i treatment substantially reduces drug costs and costs per prevented cardiovascular event, but prevents fewer events compared to a strategy of using PCSK9i treatment without bempedoic acid.

### 4.1 LDL-C target attainment

These real-world data show that only a small proportion (11.2%) of high- and very-high cardiovascular risk patients achieve their LDL-C treatment goals and combination treatments are underutilised. The low rate of LDL-C target achievement in the observation period of July 2020 to June 2021 is not substantially better compared to earlier time periods, highlighting the need for optimisation of lipid-lowering strategies [2,23,24]. The majority (>85%) of patients including the very-high risk population received moderate intensity statin monotherapy which in most cases is not enough to achieve LDL-C goals. This underscores the important opportunity to improve LDL-C lowering by combination LLT. In our simulation model, the addition of ezetimibe increased the proportion of patients with controlled LDL-C to 33%. The addition of bempedoic acid further increased goal achievement to 62%. The simulation shows that two thirds of the population would require PCSK9i treatment on top of statin and ezetimibe therapy to attain the LDL-C target. Using the bempedoic acid-based strategy, only one third of the population would need PCSK9i treatment for LDL-C target achievement. These findings are qualitatively consistent with previous simulation studies [25–27]. Another novel finding of this study is that approximately 70% of patients with high cardiovascular risk are projected to reach their LDL-C goal with the bempedoic acid-strategy. This finding is of practical importance as PCSK9i treatment is not reimbursed for most high-risk patients in contrast to patients at very-high risk.

A recent real-world study reported substantial inter-individual heterogeneity in LDL-C lowering in response to bempedoic acid treatment [28]. Although limitations such as the single-centre design and small sample size (n = 73) apply to this study, the heterogeneity is similar to observations with other oral LLT such as statins and ezetimibe [29]. Of note, the LDL-C lowering reported by Warren et al. (–36.7% at <3 months) [28] was significantly higher than the much more conservative simulation approach of our study. The inter-individual variability with LLT emphasizes the importance of follow-up measurements of LDL-C to ensure adequate response to the medication.

### 4.2 Cost considerations

Our study shows that implementing bempedoic acid into treatment algorithms to achieve LDL-C goals reduces drug costs compared to a strategy in which target achievement is realized by PCSK9i alone in patients on statin and ezetimibe. Bempedoic acid implementation is associated with 35.9% lower drug costs. PCSK9i treatment results in a more potent LDL-C reduction compared to bempedoic acid. Treating two thirds of the population with PCSK9i compared to using PCSK9i in one third in the bempedoic acid-based strategy would translate into 23% more prevented first ASCVD events. At the same time, the cost per prevented events would increase by 17.3%.

### 4.3 Strengths and limitations

Strengths of this study include using a large sample size of a contemporary and representative cohort in the German outpatient setting with longitudinal follow-up on LLT, LDL-C, and cardiovascular events. Per design, we did not include intensification of statin treatment in our simulation algorithm, however, we considered patients who were on LLT for at least four weeks and could therefore have undergone statin titration previously. In addition, statin titration may have had only small effects on LDL-C and can be associated with statin intolerance which is the main reason for the use of PCSK9i in Germany [30,31]. Including up-titration to maximal statin doses and to high-intensity statin treatments in the simulation would decrease the number of patients requiring add-on LLT without altering the main findings in terms of relative differences between the two treatment scenarios; however, the absolute numbers of additional treatment costs and prevented events would decrease with lower starting points of LDL-C on maximum statin therapy with presumably modest changes as to the already high proportion of patients on moderate-intensity statin therapy. In our simulation, bempedoic acid was stopped and replaced by PCSK9i if the LDL-C goal was not reached to reflect a strategy of reducing the number and the cost of medications. Assumptions on relative risk reduction achieved with bempedoic acid were based on the previously published CTTC meta-analyses [21,22]; the effect of lipid-lowering by bempedoic acid on cardiovascular risk reduction is being investigated directly in a global, randomised, controlled cardiovascular outcomes phase 3 trial (CLEAR Outcomes; NCT02993406) [8]. Genetic variants that mimic the effect of ATP citrate lyase inhibitors and statins appeared to lower plasma LDL-C levels by the same mechanism of action and were associated with similar decreased risk of cardiovascular disease per unit decrease in the LDL-C level [32]. The simulation is based on outpatient data that represent the majority of patients in Germany treated with LLT [2]. Lastly, patients without diagnosed hypercholesterolemia or LLT and LDL-C result were not included in this analysis.

## 5 Conclusions

Bempedoic acid is projected to substantially decrease the need of PCSK9i treatment in patients with high and very-high cardiovascular risk to attain LDL-C treatment goals. A strategy based on PCSK9i treatment without using bempedoic acid is more expensive and leads to more prevented events because of more potent LDL-C lowering. However, implementing oral bempedoic acid into the treatment algorithm after statins and ezetimibe prior to PCSK9i treatment markedly reduces drug costs and incremental drug costs per additional prevented event. This information is of high practical relevance for the very large number of patients without access to PCSK9i treatment.

## Supporting information

S1 Table Definition of statin intensity. Notes: The classification is based on the study by Fox et al. [1]. LDL-C: Low-density lipoprotein cholesterol.
(PDF)

S2 Table ICD 10 codes underlying patient selection and cardiovascular risk factor definitions. Notes: Atherosclerotic cardiovascular disease (ASCVD) was defined as at least one diagnosis of coronary artery disease, cerebrovascular disease, or peripheral artery disease. GFR: Glomerular filtration rate.
(PDF)

S3 Table ICD 10 codes underlying the primary endpoint diagnoses. (PDF)
