# Supplementary material for: Simulation study on LDL cholesterol target attainment, treatment costs, and ASCVD events with bempedoic acid in patients at high and very-high cardiovascular risk
Source: PLoS One. 2022 Oct 27;17(10):e0276898. doi: 10.1371/journal.pone.0276898 (PMC9612573; doi:10.1371/journal.pone.0276898)
Supplement: S1 Table — Notes: The classification is based on the study by Fox et al. [1]. LDL-C: Low-density lipoprotein cholesterol. (PDF) [file pone.0276898.s001.pdf]

Table S1: Definition of statin intensity

Statin	Low intensity (LDL-C reduction < 30%)	Moderate intensity (LDL-C reduction < 30% to <50%)	High intensity (LDL-C reduction ≥ 50%)
Atorvastatin	–	< 30 mg	≥ 30 mg
Fluvastatin	< 60 mg	≥ 60 mg	–
Lovastatin	< 30 mg	≥ 30 mg	–
Pitavastatin	< 1,5 mg	≥ 1,5 mg	–
Pravastatin	< 30 mg	≥ 30 mg	–
Rosuvastatin	–	< 15 mg	≥ 15 mg
Simvastatin	< 15 mg	15–59 mg	≥ 60 mg

Notes: The classification is based on the study by Fox et al. [1].
LDL-C: Low-density lipoprotein cholesterol.

Reference:

1. Fox KM, Tai M-H, Kostev K, Hatz M, Qian Y, Laufs U. Treatment patterns and low-density lipoprotein cholesterol (LDL-C) goal attainment among patients receiving high- or moderate-intensity statins. Clin Res Cardiol. 2018; 107:380–8. doi: 10.1007/s00392-017-1193-z PMID: 29273856.
