# Supplementary material for: Simulation study on LDL cholesterol target attainment, treatment costs, and ASCVD events with bempedoic acid in patients at high and very-high cardiovascular risk
Source: PLoS One. 2022 Oct 27;17(10):e0276898. doi: 10.1371/journal.pone.0276898 (PMC9612573; doi:10.1371/journal.pone.0276898)
Supplement: S2 Table — Notes: Atherosclerotic cardiovascular disease (ASCVD) was defined as at least one diagnosis of coronary artery disease, cerebrovascular disease, or peripheral artery disease. GFR: Glomerular filtration rate. (PDF) [file pone.0276898.s002.pdf]

Table S2: ICD 10 codes underlying patient selection and cardiovascular risk factor definitions

Diagnosis, risk factor	ICD 10 code
Hypercholesterolaemia	E78.0, E78.2, E78.4, E78.5, E78.8, E78.9
Coronary artery disease	I20–I25, Z95.5, Z95.1
Cerebrovascular disease	I63, I64, G45
Peripheral artery disease	I73.9, I70, I74, E10.5, E11.5, E14.5
Diabetes mellitus	E10–E14
Diabetes mellitus with target organ damage	E10.2, E11.2, E14.2, N19 (microalbuminuria)<br>E10.3, E11.3, E14.3 (retinopathy)<br>E10.4, E11.4, E14.4 (neuropathy)
Chronic kidney disease (CKD)	GFR <30 ml/min/1.73m^2 or N18.4, N18.5 (severe CKD)<br>GFR 30–59 ml/min/1.73m^2 or N18.3 (moderate CKD)
Hypertension	I10–I15
Smoking	Z72.0, Z71.6, F17

Notes: Atherosclerotic cardiovascular disease (ASCVD) was defined as at least one diagnosis of coronary artery disease, cerebrovascular disease, or peripheral artery disease. GFR: glomerular filtration rate.
