# Supplementary material for: Simulation study on LDL cholesterol target attainment, treatment costs, and ASCVD events with bempedoic acid in patients at high and very-high cardiovascular risk
Source: PLoS One. 2022 Oct 27;17(10):e0276898. doi: 10.1371/journal.pone.0276898 (PMC9612573; doi:10.1371/journal.pone.0276898)
Supplement: S3 Table — (PDF) [file pone.0276898.s003.pdf]

Table S3: ICD 10 codes underlying the primary endpoint diagnoses

Diagnosis	ICD 10 code
Cardiovascular death	I46.1
Non-fatal myocardial infarction	I20–I25
Non-fatal stroke	I63, I64, G45
Coronary revascularization	Z95.5, Z95.1
